# Immediate Implant Placement and Provisionalization in the Esthetic Zone: A 6.5-Year Follow-Up and Literature Review

**DOI:** 10.1155/2021/4290193

**Published:** 2021-09-15

**Authors:** Hsiu-Wan Meng, Esther Yun Chien, Hua-Hong Chien

**Affiliations:** ^1^Department of Periodontics and Dental Hygiene, The University of Texas Health Science Center School of Dentistry, Houston TX, USA; ^2^Private Practice, Columbus OH, USA; ^3^Division of Periodontology, College of Dentistry, The Ohio State University, Columbus OH, USA

## Abstract

The success of dental implant therapy in the esthetic zone requires not only functional osseointegration but also a satisfactory esthetic outcome. To establish harmony, balance, and continuity of gingival architecture between an implant restoration and the adjacent natural dentition is challenging. Immediate implant placement and provisionalization following tooth extraction have been documented as a predictable treatment modality, with fewer surgical interventions needed, to replace a missing tooth in the esthetic zone. This case report illustrates immediate implant placement and provisionalization to replace a failing maxillary right central incisor while maintaining optimal gingival esthetics. The maxillary right central incisor was extracted without flap elevation to minimize soft and hard tissue trauma. Immediately afterwards, the implant was installed using a surgical stent and restored with a provisional crown that had no occlusal contacts. During healing, no significant adverse effects were observed clinically or radiographically. This proposed treatment modality provided the patient with immediate esthetics, function, and comfort without any complications during a follow-up period of 6.5 years.

## 1. Introduction

Dental implants have become a viable treatment option for completely and partially edentulous patients. Since 1968 when Dr. Branemark first introduced the concept of osseointegration, a vast amount of clinical investigation with long-term follow-up has been conducted on the effectiveness and predictability of implant-supported restorations [1–3].

Implants can be placed using a two-stage or one-stage procedure. Traditionally, they are placed using a two-stage procedure, where they are initially completely submerged under mucosal tissue during the healing process to avoid any functional loading and then later uncovered in a second minor surgery. Two-stage implants installed using a one-stage procedure have been demonstrated to be as predictable as those placed following the traditional two-stage procedure [4]. Furthermore, a prospective clinical study on two-stage implants placed using a one-stage procedure versus one-stage implants has shown comparable clinical and radiographic outcomes during the healing period [5]. Therefore, more and more implants are now placed using a one-stage procedure, in which the healing abutment is connected to the implant fixture at the time of implant surgery. A systematic review by Esposito et al. [6] looked at 5 randomized controlled trials of adults with a minimum follow-up of 6 months after loading. They found no significant differences in measured outcomes between one- and two-stage implant placement techniques. Moreover, the development of implant surface characteristics has increased bone-to-implant contact and implant stability at earlier stages in the healing process. Ultimately, the conventional protocol of including a healing period of 6 months for the maxilla and 4 to 6 months for the mandible has been reduced significantly. Some specific implant surface characteristics allowed functional loading 6 weeks after implant placement, which is a major breakthrough in surface technology [7].

Immediate implant placement in a fresh extraction socket has been reviewed extensively in the literature [8, 9]. Traditionally, a 3-month waiting period after tooth extraction is required for soft and hard tissue healing before placing a dental implant. The protocol of placing an implant in a healed socket and subsequently restoring the implant with a prosthesis is recognized as a highly predictive treatment modality for partially edentulous patients [10]. Recently, the traditional protocol has been increasingly replaced by a faster protocol in which an implant is placed immediately in a fresh extraction socket in combination with immediate restoration [11].

Immediate implant placement and restoration of a single implant in the esthetic zone have several proposed benefits, including reduced overall treatment time, fewer surgical procedures, less traumatic surgery, and greater patient satisfaction [12]. On the other hand, this treatment protocol also has inherent disadvantages, such as difficulty in achieving implant stability, higher risk of implant failure, unpredictable soft and hard tissue levels, and the need for bone grafts [13]. The placement of a provisional restoration on a single implant in the esthetic zone has been advocated for creating a good soft tissue contour, especially in conjunction with immediate implant placement [14]. Immediate implant placement and provisionalization can be a predictable treatment modality to replace a hopeless tooth in the esthetic zone when primary implant stability can be achieved, and the provisional restoration can be adjusted to clear all centric and eccentric contacts.

## 2. Case Presentation

**Figure 1:**
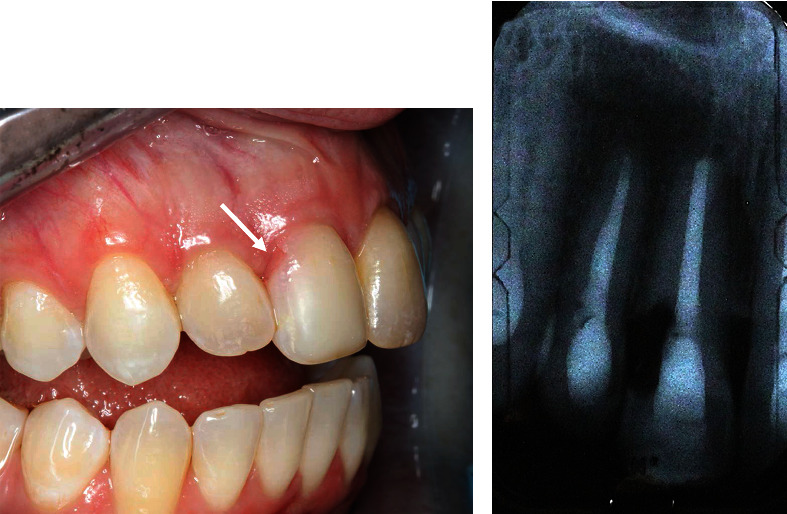
(a) Upper right central incisor with external root resorption (white arrow) was confirmed by (b) a periapical radiograph.

A 38-year-old Caucasian female reported to the Graduate Periodontics Clinic at the College of Dentistry, The Ohio State University, for a single tooth extraction and implant therapy. A nonrestorable maxillary right central incisor with external root resorption (Figure 1) was observed at the initial examination and was confirmed by a periapical film. The patient reported a history of trauma around the upper anterior teeth resulting in endodontic treatment of teeth 12, 11, and 21. She reported no significant medical history except an allergy to penicillin. She had no history of smoking and was classified as American Society of Anesthesiologists (ASA) Physical Status Classification System I. A comprehensive periodontal exam revealed no probing depths greater than 3 mm, and the embrasure spaces between the maxillary incisors were completely filled with interdental papilla. Neither tooth mobility nor percussion/palpation pain was detected in teeth 12 to 22. Radiographic examination illustrated external root resorption on the distal aspect of tooth 11 at the CEJ level with no apical radiolucency. After clinical and radiographic preoperative analysis to assess the patient risk profile for immediate implant placement, four treatment options (immediate implant placement, early implant placement with soft tissue healing, early implant placement with partial bone healing, and late implant placement) [15] were reviewed with the patient. Immediate implant placement with provisionalization after tooth extraction was recommended, and the patient accepted the treatment plan.

**Figure 2:**
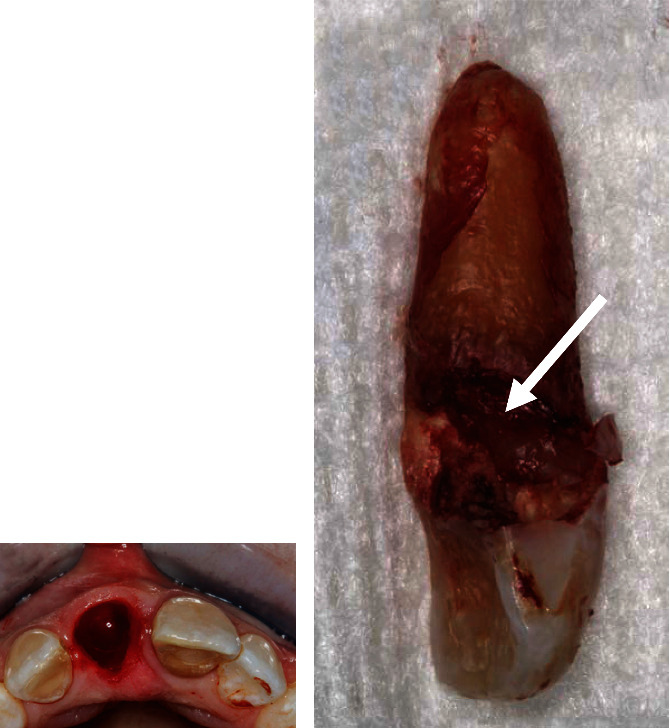
(a) Extraction socket of tooth 11 after minimally invasive tooth extraction without flap elevation. (b) External root resorption was observed on the distal surface of the extracted upper right central incisor (white arrow).

**Figure 3:**
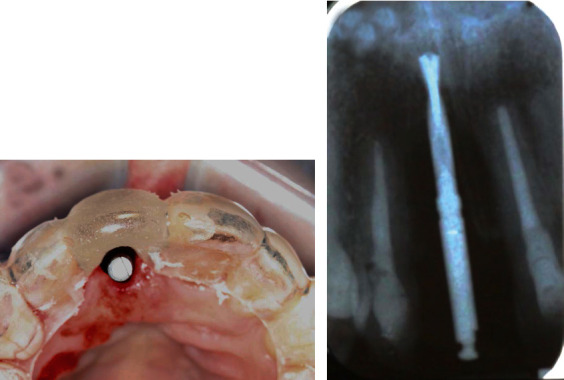
(a) Initial implant osteotomy was made at the cingulum region using a precision drill under the guidance of a surgical stent. (b) A periapical radiograph was taken with a 2 mm twist drill to verify the depth and angulation for implant placement.

**Figure 4:**
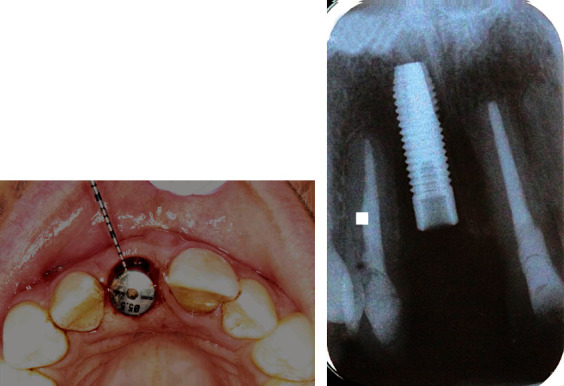
(a) A 5.5 mm diameter healing abutment was connected to an OsseoSpeed 4.0 × 15 mm implant at the position of tooth 11. (b) A periapical radiograph was taken to validate that the implant shoulder was located at least 3 mm apical to the CEJ of the adjacent teeth.

**Figure 5:**
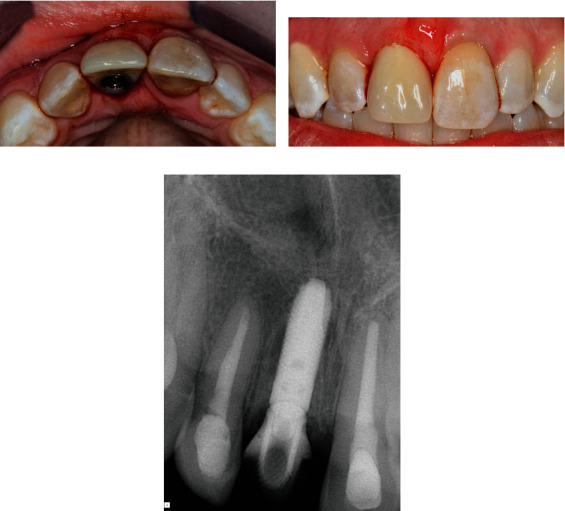
A customized screw-retained provisional crown was made chairside immediately after implant placement. (a) Occlusal view of the provisional crown showing the location of the implant at the cingulum region. (b) Facial view of the provisional crown displaying its subgingival contour conformed to support the soft tissue emergence profile. (c) A periapical radiograph was taken immediately after provisional crown restoration.

One hour before the surgery, the patient was given 600 mg of Clindamycin for surgical prophylaxis to enhance the success rate of the implant [16]. Under local anesthesia, an intrasulcular incision was placed 360° around tooth 11 using a 15C blade to cut the connective tissue fibers above the bone. An anterior periotome was used to sever the periodontal ligament to facilitate the removal of tooth 11 with minimal damage of the surrounding alveolar bone. The tooth was then removed using extraction forceps with controlled rotational force without flap elevation. Extreme care was taken to avoid fracturing the socket walls, especially the buccal plate, so the gingival and bone architecture would be preserved. The external root resorption was also confirmed on the tooth removed (Figure 2). The extraction socket was thoroughly debrided using a serrated excavator to remove any granulation tissue and then irrigated with sterile saline before osteotomy. A periodontal probe was used to confirm the integrity of the socket walls and to verify that the distance from the alveolar crest to the gingival margin was less than 3 to 4 mm as recommended for immediate implant placement. A sharp precision drill (Nobel Biocare, Yorba Linda, CA) was used to penetrate the palatal wall of the extraction socket that guided the initial preparation of an osteotomy. The osteotomy was prepared under the guidance of a surgical stent and extended about 3 to 5 mm beyond the root apex to ascertain implant primary stability [17]. A periapical radiograph was taken with a twist drill to verify the angulation (Figure 3). An OsseoSpeed 4.0 × 15 mm implant (Astra Tech Dental) was placed immediately into the fresh extraction socket following the rule of restorative-driven 3-dimensional placement [18, 19], and the implant shoulder was positioned at least 3 mm apical to an imaginary line connecting the cementoenamel junctions of the adjacent teeth [20, 21] (Figure 4). The primary stability of the implant was confirmed by achieving a torque resistance of 40 Ncm. Hydrated freeze-dried bone allograft was placed in the gap between the labial bony wall and the implant. A customized screw-retained provisional crown was made so its subgingival contour supported the soft tissue emergence profile and protected the blood clot and graft particles (Figure 5). The temporary crown was torqued to 25 Ncm and left to heal for 9 months. The occlusion of the provisional crown was adjusted to ensure that it was free from any contact during articulation. The patient was informed that she should not place any pressure on the provisional crown during the healing period. Postoperatively, Clindamycin 300 mg was given four times a day for 7 days and ibuprofen 600 mg every 4 to 6 hours for the control of infection and pain, respectively. Chlorhexidine gluconate 0.12% oral rinses were prescribed twice daily for 2 weeks. The patient was advised to adhere to a soft diet and avoid forces on the anterior teeth.

**Figure 6:**
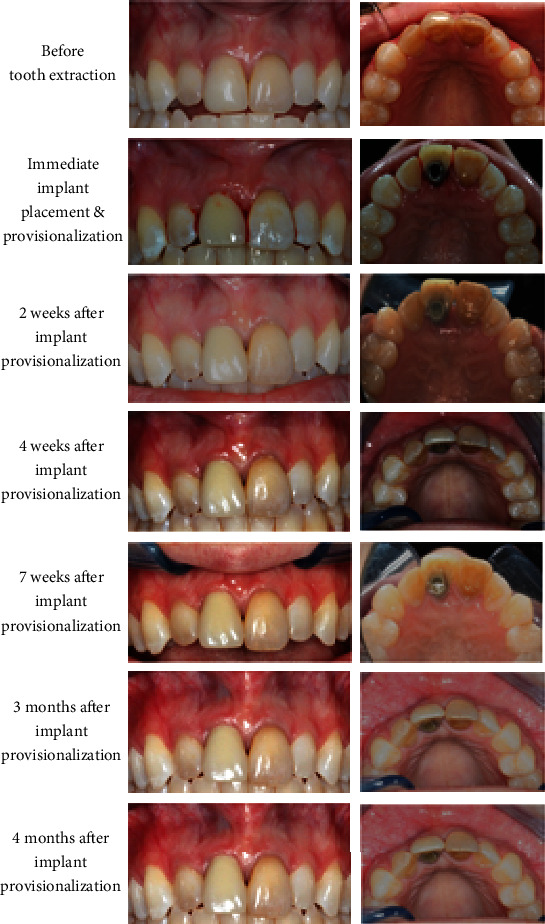
Clinical photos of tooth 11 before and after immediate implant provisionalization. During the 4-month follow-up period, the gingival tissue around implant was pink and healthy. The patient was satisfied with the esthetic outcome of the immediate implant provisionalization.

The patient received regular check-ups at 2, 4, and 7 weeks and 3 and 4 months after the implant placement surgery with no tenderness reported or adverse events observed. During the postoperative follow-up appointments, the gingival margin around the implant provisional crown appeared stable and there was functional attachment (Figure 6). After a 9-month healing period, the provisional crown was replaced by a definitive restoration. The patient was followed for 6.5 years after final crown restoration.

**Figure 7:**
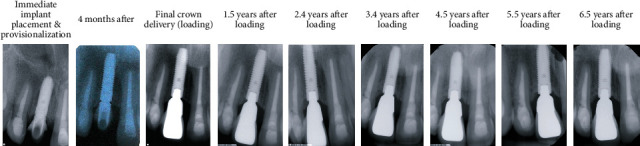
Periapical radiographs taken at immediate implant provisionalization and at various times during the 6.5-year recall period.

**Figure 8:**
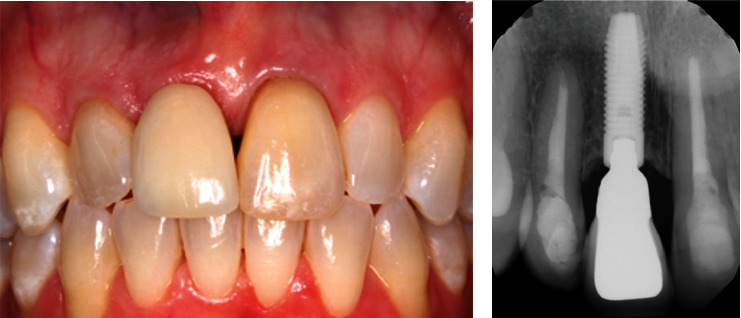
(a) Clinical photo and (b) periapical radiograph of the implant at 6.5 years after loading.

Recall appointments were made every 6 months after implant loading. At these recall appointments, the implant remained stable and functioned well without any problems. The peri-implant gingiva was pink and healthy with probing depths all within 4 mm without bleeding on probing. In addition, a periapical radiograph was taken every year for the assessment of marginal bone level on the mesial and distal sides of the implant. The radiograph revealed a stable marginal bone level around the implant (Figure 7) compared to the radiograph taken at final crown delivery, without any peri-implant radiolucency noted. An excellent esthetic outcome with patient satisfaction through immediate implant placement and provisionalization was observed during the 6.5-year follow-up period (Figure 8).

## 3. Discussion and Conclusion

Immediate implant placement refers to the placement of an implant on the day of tooth extraction and within the same surgical procedure [22]. Wohrle was the first to report the protocol for immediate implant placement and provisionalization in the esthetic zone [23], which subsequently has been adopted in numerous studies and found to be an excellent treatment modality with high success/survival rate and stable gingival architecture [17]. A systematic review on immediate implant placement indicated that most studies evaluated the success/survival rate and radiographic marginal bone levels around immediate implant placement and provisionalization [9]. However, few studies assessed soft tissue parameters such as proximal papilla levels and facial gingival levels [14, 24–36]. Lang et al. [9] reported that the estimated 2-year survival rate of implants placed immediately in fresh extraction sockets was 98.4%. Another systemic review conducted by Slagter et al. specifically investigated immediately placed single-tooth implants in the esthetic zone and also found a similar survival rate of 97.1% [37].

An extraction socket classification system for the maxillary anterior teeth, based on the soft and hard tissue components, was introduced in 2008 [38]. According to this classification system, the extraction socket in the present case was graded type I, which was suitable for immediate implant placement. Immediate implant placement and provisionalization in a fresh extraction socket is challenging and requires careful case selection [39]. In general, the soft tissue contour of the extraction socket should closely mimic that of adjacent natural teeth without vertical soft tissue deficiency [40, 41]. Furthermore, the keratinized gingival width on the midbuccal aspect of the socket should be ≥2 mm, with a thick gingival biotype (≥2 mm). The apical bone beyond the extraction socket should be ≥4 mm to achieve primary stability of the implant. The tip of the mesial and distal papillae should lie between the interdental contact and the most coronal extent of interproximal CEJ. In other words, the papilla appearance should be categorized as Class I based on the classification system described by Nordland and Tarnow [42]. Regarding the hard tissue in the fresh extraction socket, the distance between the osseous crest and the gingival margin should be ≤3 mm on the midfacial and ≤4.5 mm on the proximal aspects [39]. In addition, the distance from the facial bone of the extraction socket to the implant should be ≥2 mm to maintain the implant soft tissue profile for ideal esthetics.

In the present case, the implant was positioned at the center of the final restoration with a clearance of ≥1.5 mm between the implant fixture and the adjacent teeth to minimize the risk of damaging adjacent natural teeth. Furthermore, the implant was also placed in the cingulum position with ≥2 mm between the facial bone and the implant fixture. A recent animal study on dogs suggested a critical buccal bone wall thickness of at least 1.5 mm to compensate for the dimensional changes occurring after implant placement and the progression of peri-implantitis [43]. To obtain a better emergence profile, the implant platform should be located apicocoronally at least 3 mm from the cementoenamel junction of the adjacent tooth [39]. According to the classification system described by Kan et al. [44] on sagittal root position in relation to the anterior maxillary osseous housing, the sagittal root position in the present case was classified as Class I, where a considerable amount of bone was present on the palatal aspect, indicating a favorable setting for immediate implant placement. About 81% of teeth were classified as Class I in Kan et al.'s study.

Placing the implant in the cingulum position often results in a gap between the implant and the labial cortical plate. Dramatic changes in ridge dimension following tooth extraction have been demonstrated in clinical and histological studies, and bone augmentation has been effective in promoting bone fill and defect resolution at immediate implant sites [45, 46]. Clinical and histologic studies have shown that an esthetic hard tissue contour can be maintained both vertically and horizontally when the implant-socket gap is filled with bone grafting materials [47, 48]. Therefore, the implant-socket gap was filled with freeze-dried bone allograft in this case and the marginal bone level changes were negligible as indicated by periapical radiographs taken 6.5 years after loading.

Chu et al. proposed the dual-zone concept where bone grafts were placed in the bone and tissue zones to serve as a scaffold to maintain hard and soft tissue volume and the blood clot to facilitate initial healing [49]. Xenografts were placed in the gap between the implant and buccal bone until the gap was filled to the most coronal aspect of the free gingival margin. The authors suggested that xenograft particles incorporated into the soft tissue when placing the provisional restoration can minimize ridge collapse and increase peri-implant soft tissue thickness [50, 51]. These studies were followed for 6 months to 4 years after delivery of the definitive tooth restoration, and further long-term studies are still needed. In our case, the concept was not utilized at the time of surgery. We placed bone grafts in the gap between the implant fixture and buccal plate but did not fill it all the way up to the soft tissue level; nevertheless, our 6.5-year follow-up showed stable hard and soft tissue results.

The most challenging goal for implant therapy in the esthetic region is achieving soft and hard tissue stability over time. Midfacial mucosal recession has been one of the most commonly reported complications following immediate implant placement [52]. In the present case, multiple factors were considered to avoid a compromised outcome, and no recession was noted during 6.5 years of follow-up. It has been documented in the literature that anatomic factors associated with midfacial recession following immediate implant placement are the gingival biotype and the width of the keratinized mucosa [53]. More recession has been observed on implants placed in patients with a thin gingival biotype and narrow keratinized mucosa with a width less than 2 mm. A damaged facial bone wall also represents a significant risk factor for midfacial recession. Kan et al. reported that midfacial recessions greater than 1.5 mm were observed one year after immediate implant placement and provisionalization in 8.3% of tooth sockets with a narrow V-shaped facial bony defect exceeding 3 mm despite simultaneous guided bone regeneration and soft tissue grafting [26]. Furthermore, midfacial recession increased to 42.8% and 100% when the bony defect extended wider onto the mesial or distal aspect of the failing tooth (U-shaped) or adjacent teeth (UU-shaped), respectively. Staged reconstruction of unfavorable U- and UU-shaped facial bony defects followed by delayed implant placement was strongly recommended. Additionally, the position of the implant has consistently played an important role in the midfacial mucosal level [54]. Placing implants too buccally has been associated with more recession of the midbuccal mucosa. Immediate implant placement using a flapless approach also demonstrated an average of 0.89 mm less midfacial mucosal recession at 1 year after implant placement [55] compared to open-flap implant placement. Instant provisionalization of immediate single-tooth implants has also been shown to have 0.75 mm less midfacial recession in comparison with delayed restoration after 1 year [29].

In the present case, the patient was given 600 mg of Clindamycin one hour preoperatively followed by Clindamycin 300 mg four times a day postoperatively for 7 days. A Cochrane systemic review, including 6 randomized controlled clinical trials, assessed the effects of systemic prophylactic antibiotics at dental implant placement and found that there was no clear evidence whether postoperative antibiotics were beneficial. However, the routine use of a single dose of 2 g or 3 g prophylactic amoxicillin one hour preoperatively significantly reduces failure of dental implants [56]. On the other hand, a systematic review by Lang et al. [9] investigated 33 prospective studies where antibiotics were prescribed in immediate implant placement cases and compared subjects who had received a preoperative single dose of antibiotic prophylaxis (PreOP), postoperative antibiotic use of 5-7 days (PostOP), and a single dose preoperatively plus 5-7 days of postoperative antibiotics (PreOP + PostOP). Lower estimated annual failure rates were found in groups that were provided with a course of postoperative 5-7-day antibiotics (0.51% and 0.75% in PostOP and PreOP + PostOP, respectively, compared to 1.87% in PreOP). It was concluded that the regimen of antibiotic use affected the survival rate significantly as the duration of usage might have played an important role.

Similar clinical outcomes were found for submerged and nonsubmerged implants placed in fresh extraction sockets [57], indicating that there is no need to submerge the immediate implant with primary closure compared to a nonsubmerged approach with healing abutments. Likewise, comparable outcomes with regard to implant success rates and radiographic bone level stability were also observed for both immediate and delayed loading of immediately placed implants [58]. Therefore, submerging an immediate implant with primary closure did not lead to significant benefits over a nonsubmerged approach with immediate provisionalization. Furthermore, it is worth noting that the nonsubmerged approach demonstrated better attached gingiva and mucogingival junction (MGJ) harmony and fewer soft tissue adverse effects [57, 58]. Because primary closure results in coronal displacement of the MGJ and a decrease in the amount of buccal attached gingiva, apically positioned flaps at implant uncovering or further mucogingival surgery may be necessary to correct this problem.

Although only two-dimensional radiographs (periapical and panoramic) were taken before implant placement for the assessment of bone quantity, we strongly recommend the utilization of state-of-the-art technology such as virtual implant planning systems that integrate CBCT data to assess bone quantity and allow for greater predictability and accuracy of implant placement in the esthetic zone. Current technology also includes a digital workflow for computer-guided implant surgery which improves diagnostic capabilities and provides more precise and prosthetically driven planning and implant placement [59]. However, substantial errors can occur at each individual step and can accumulate, significantly impacting the final accuracy with potentially disastrous deviations from ideal implant placement [60]. It is always crucial to mitigate these risks through complete understanding of the guided implant surgery process, thorough and careful surgical technique, advanced comprehensive training, and adequate case preparation.

Above all, primary stability of the implant is the most important factor for immediate implant provisionalization. The torque at time of implant placement can be used as an indicator of initial stability. Ottoni et al. reported that an insertion torque greater than 32 Ncm is necessary for an implant restored with an immediate provisional crown [61]. They found that 9 out of 46 implants failed because they had an insertion torque of 20 Ncm. In the present case, the primary stability of implant was achieved with an insertion torque > 35 Ncm.

The purpose of this case report was to review the surgical steps of immediate implant placement and provisionalization following tooth extraction. Careful planning and case selection are critical to ensure implant success with satisfactory esthetic outcomes. Immediate implant placement and provisionalization appear to add significant advantages for the esthetically driven replacement of anterior teeth. This treatment strategy helps to preserve physiologic soft and hard tissue architecture and, therefore, predictably leads to excellent esthetic outcomes and patient satisfaction. The long-term success of this approach depends on the achievement of primary stability, and the immediate provisionalization must be designed to avoid any centric and eccentric contact during healing. Extraction using a flapless procedure followed by immediate implant placement and provisionalization without functional loading offers a predictable therapeutic option for single-tooth replacement in the esthetic zone.

## Data Availability

The data used to support the findings of this study are included within the article.
